# Importance of colour vision testing in school based eye health examination

**Published:** 2017

**Authors:** Asitkumar Jadhav, Prem Kumar SG, Sabitra Kundu

**Affiliations:** Senior Optometrist, Programme Development, Mission for Vision, Mumbai, India; Manager, Research, Mission for Vision, Mumbai, India; Head, Programme Development, Mission for Vision, Mumbai, India

**Figure:**
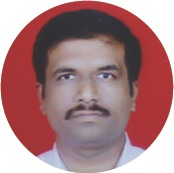
Asitkumar Jadhav

**Figure:**
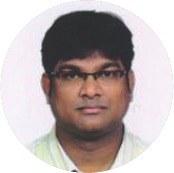
Prem Kumar SG

**Figure:**
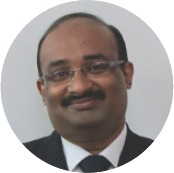
Sabitra Kundu

**It is recommended that, in the context of rapid improvements in the educational needs of the children, which are currently more inclined towards colour-based learning, the government should make efforts in formulating policies and guidelines for comprehensive school eye health programmes to screen children for colour vision deficiencies.**

**Figure:**
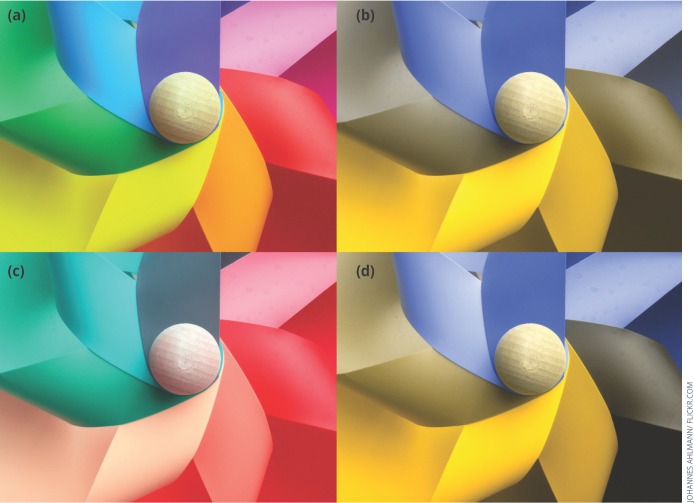
Comparison between the types of blindness; (a) Normal colour vision; (b) green-blindness (deuteranopia); (c) blue-blindness (tritanopia); (d) red-blindness (protanopia).

## What is colour vision?

Colour vision is the ability of the eye to detect different wavelengths of light and to distinguish between these different wavelengths and their corresponding colours.^1^ The Young Helmholtz theory of trichromatic colour vision, postulates the existence of three kinds of cones, each containing a different photo pigment which is maximally sensitive to one of the three primary colours. Normal, or trichromatic, colour vision is mediated by three types of cone photoreceptors – designated short- (S), middle- (M), and long- (L) wavelength-sensitive, showing peak absorbencies at light wavelengths of 415 nanometres (nm), 530 nm and 560 nm, respectively. Blue, green and red are thus called primary colours as any colour can be produced by mixing appropriate proportion of these three colours.^2^

## What is colour vision deficiency?

Colour Vision Deficiency (CVD) is the inability to distinguish certain colours. The defects in colour vision result from the absence, malfunction, or alteration of one, two or all of the photo-pigments. There are broadly two types of CVD:

- total colour blindness and
- partial colour blindness.

Partial colour blindness is again sub-classified as red–green and blue-yellow.^3^,^4^ Impairment in colour vision can be either hereditary or acquired. Many people are affected by colour blindness, but many of them remain undetected as they simply adapt to the environment.^3^ The prevalence of CVD has been studied in various population groups around the world, with the prevalence in most populations reported to be from 2% to 10% for boys and less than 0.1% to 3% for girls.^5^,^6^

**Figure:**
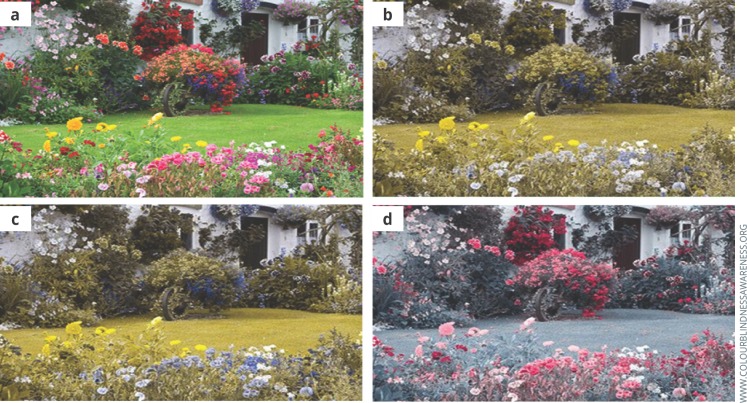
(a) Normal vision; (b) Deuteranopia; (c) Protanopia; (d) Tritanopia.

## Impact of colour vision deficiency on children

Most people with colour vision defects develop effective adaptive strategies and behaviours, and they use other clues, such as a colour's saturation, to deal with any potential limitations in their professional and personal lives. Increasing use of colour in education has raised concerns for children with CVD, but robust evidence is lacking.^7^ Children with CVD may perform poorly on tests or assignments that employ color-coded materials. If the student and their parents are unaware of the issue, those students may struggle in class, leading teachers to group them in the wrong academic track at school. Dr. Varma and other researchers from the Multi-Ethnic Paediatric Eye Disease Study Group tested 4,005 Californian preschool children, aged three to six years in Los Angeles and Riverside counties for colour blindness. Their findings suggest that successful colour vision deficiency testing can be done starting at age four.^8^ Prof. Chandak and colleagues recently reported on 850 children aged 10 to 15 years in Wardha district of Maharashtra, India emphasising the importance of early diagnosis of this dysfunction in children that would enable them to adapt well and better plan for their future, professional lives.^9^ Cole stressed that school children should know if they have CVD so they can be helped more quickly to find adaptive strategies and be able to take it into account when planning their career.^10^ Evidence from other studies in India suggests higher prevalence of this condition in certain pockets of the country.^11^

Screening of colour vision defects are relatively quick and easy. The Ishihara charts are the most widely used in India among children that help diagnose the type of deficiency and its severity, although emphasis on computer-based approaches have been recommended recently.^12^ Screening children for these disorders is an established practice in the United Kingdom and the rest of the western world. This is so that those affected can be advised about occupational preclusions such as driving, aviation, art, photography, jewellery, tailoring, fashion design, defence services, engineering and medicine. However, there are no such colour vision screening guidelines or policies available in India.

Furthermore, robust scientific evidence on CVDs amongst the Indian populations is scanty. In the absence of standard guidelines in our country, parental education, awareness, genetic testing and counselling strategies in the regions with high CVD prevalence, could help children and their families cope with the condition and plan their respective professional futures. It is recommended that, in the context of rapid improvements in the educational needs of the children, which are currently more inclined towards colour-based learning, the government should make efforts in formulating policies and guidelines for comprehensive school eye health programmes to screen children for CVDs.
